# The Devon Active Villages Evaluation (DAVE) trial of a community-level physical activity intervention in rural south-west England: a stepped wedge cluster randomised controlled trial

**DOI:** 10.1186/s12966-014-0094-z

**Published:** 2014-07-18

**Authors:** Emma Solomon, Tim Rees, Obioha C Ukoumunne, Brad Metcalf, Melvyn Hillsdon

**Affiliations:** 1 Sport and Health Sciences, College of Life and Environmental Sciences, University of Exeter, St Luke’s Campus, Heavitree Road, Exeter EX1 2 LU, UK; 2 PenCLAHRC, University of Exeter Medical School, Veysey Building, Salmon Pool Lane, Exeter EX2 4SG, UK

**Keywords:** Physical activity, Stepped wedge cluster randomised controlled trial, Community-level intervention, Rural communities

## Abstract

**Background:**

The majority of adults are not meeting the guidelines for physical activity despite activity being linked with numerous improvements to long-term health. In light of this, researchers have called for more community-level interventions. The main objective of the present study was to evaluate whether a community-level physical activity intervention increased the activity levels of rural communities.

**Methods:**

128 rural villages (clusters) were randomised to receive the intervention in one of four time periods between April 2011 and December 2012. The Devon Active Villages intervention provided villages with 12 weeks of physical activity opportunities for all age groups, including at least three different types of activities per village. Each village received an individually tailored intervention, incorporating a local needs-led approach. Support was provided for a further 12 months following the intervention. The evaluation study used a stepped wedge cluster randomised controlled trial design. All 128 villages were measured at each of five data collection periods using a postal survey. The primary outcome of interest was the proportion of adults reporting sufficient physical activity to meet internationally recognised guidelines. Minutes spent in moderate-and-vigorous activity per week was analysed as a secondary outcome. To compare between intervention and control modes, random effects linear regression and marginal logistic regression models were implemented for continuous and binary outcomes respectively.

**Results:**

10,412 adults (4693 intervention, 5719 control) completed the postal survey (response rate 32.2%). The intervention did not increase the odds of adults meeting the physical activity guideline (adjusted OR 1.02, 95% CI: 0.88 to 1.17; P = 0.80), although there was weak evidence of an increase in minutes of moderate-and-vigorous-intensity activity per week (adjusted mean difference = 171, 95% CI: -16 to 358; P = 0.07). The ineffectiveness of the intervention may have been due to its low penetration—only 16% of intervention mode participants reported awareness of the intervention and just 4% reported participating in intervention events.

**Conclusions:**

A community-level physical activity intervention providing tailored physical activity opportunities to rural villages did not improve physical activity levels in adults. Greater penetration of such interventions must be achieved if they are to increase physical activity prevalence at the community level.

**Trial Registration:**

Current Controlled Trials ISRCTN37321160.

## Background

Leading a physically active lifestyle reduces the risk of all-cause mortality, cardiovascular disease, type two diabetes, and some cancers, and can improve musculoskeletal health, control body weight, and reduce symptoms of depression [[1]]. In order to achieve such benefits, adults are recommended to undertake a minimum of 150 minutes of at least moderate-intensity physical activity per week [[2],[3]]. Despite this, in the Health Survey for England 2008, only 39% of men and 29% of women reported doing sufficient physical activity [[4]]. Based on this evidence, interventions to increase physical activity levels are now considered to be as important to population health as interventions to lower tobacco use or reduce blood pressure [[3]]. Fortunately, substantial health benefits can be achieved through relatively modest changes in physical activity among large segments of the population [[5]].

Physical activity is a complex behaviour determined by the interaction of a large number of personal, social, and environmental factors [[6]–[8]]. In order to change population prevalence, interventions need to be both effective and reach large numbers of people. The majority of physical activity interventions have been delivered at the level of the individual, aimed at changing personal behaviour [[9]], whereas it is community-level interventions that have the potential to produce long-lasting benefits for the whole community [[10]]. To date, evaluations of community-level interventions have typically used weak study designs, such as uncontrolled, pre-post evaluations, and are therefore unable to attribute any observed changes to the intervention [[11]]. A ‘Behaviour Change’ report by the House of Lords [[9]] noted that pragmatic community-level interventions funded by public money are routinely delivered with little or no evaluation. The report stated that there is no excuse for weak evaluations, with the recommendation that rigorous evaluation plans should be in place before interventions are funded [[9]]. Although randomised controlled trials are considered the most powerful design for evaluating interventions [[12]], they tend to focus on individuals rather than communities, such that the findings of traditional randomised controlled trials are not always applicable in the real world [[13]]. In contrast, cluster randomised trials, which randomise groups (e.g., communities) and measure outcomes on individuals within those groups, may be more appropriate for evaluating interventions that are by necessity delivered to groups rather than individuals [[9],[14]]. As an alternative to the traditional parallel groups design, in which clusters are randomised to either an intervention or control arm, the stepped wedge trial design [[15]] allows the staggered delivery of an intervention to all trial clusters over a number of time periods, with clusters crossing over from the control to intervention arm. Stepped wedge designs are beneficial when an intervention cannot be delivered to many clusters at the same time, or when it would be unethical to withhold the intervention because it is strongly believed the intervention will do more good than harm [[16]].

Although 20% of the English population (approximately 10 million people) live in non-urban locations [[4]], rural populations are generally understudied [[17],[18]]. Studies examining the influence of residential location on physical activity have generally found that rural adults are less likely than urban adults to meet recommended activity guidelines, suggesting rural residents are appropriate targets for future physical activity interventions [[19]–[23]]. Compared to their urban counterparts, rural residents are more likely to report lower social support and limited access to exercise facilities as barriers to being physically active [[20],[21]]. Other barriers reported by rural women include the remoteness of the environment they live in, how rural the local area is [[24]], and being too far away from activity facilities [[25],[26]]. It is clear that rural populations face a unique set of challenges associated with physical activity behaviour, and yet they have received very little research attention to date, especially in the United Kingdom. The aim of the present research was to evaluate the effectiveness of a community-level physical activity intervention—*Devon Active Villages*—using a stepped wedge cluster randomised controlled trial design.

## Methods

The data presented are from a stepped wedge cluster randomised controlled trial design evaluating Devon Active Villages*,* a community-level physical activity intervention in south-west England. The study design and sampling have been described in detail elsewhere [[27]].

### Participants

**Figure 1:**
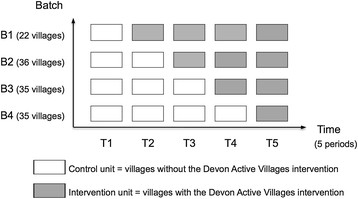
**Design of the DAVE study. One batch (B1, B2, B3, B4) represents one group of intervention villages.** Each time period (T1, T2, T3, T4, T5) represents a data collection point. Each unit (control or intervention) represents one time period of one batch.

The research took place in the seven rural regions of Devon, south-west England. Villages with populations of 500–2000 people formed the sampling frame for the intervention. The range of eligible population sizes were set so that villages were large enough to have local facilities suitable for physical activity, but limited in the amount of activity opportunities they could offer. The first period (stage) took the form of a baseline period, where no villages received the intervention. The intervention was administered sequentially to 128 villages over the subsequent four time periods (Figure 1). The time period in which villages first received the intervention was randomised (stratified by region) using computer generated random numbers. The number of villages that were to receive the intervention at each period in each village was pre-specified by Active Devon, placing further restriction on the allocation sequence. Twenty-two villages received the intervention in the second period (April-June 2011), 36 in the third period (September-November 2011), 35 in the fourth period (April-June 2012), and 35 in the fifth period (September-November 2012).

Data collection for the evaluation took the form of a postal survey conducted at five fixed time points: baseline (in the month prior to commencement of the first intervention period) and within a week of the end of each of the four intervention periods. A repeated cross-sectional design was employed, in which a random sample of households within each cluster was selected to receive the survey at each period. The addresses of all households in participating villages were purchased from a private company (Address List Utility, Arc en Ciel, Version 3.1 PAF Quarter 1, 2011), and the order in which households were approached to participate in the survey at each period was randomly generated. Households were sent a questionnaire, a participant information sheet, and a prepaid return envelope. The adult in each household who had most recently had a birthday was invited to complete the survey. Eligible participants were aged 18 years or over and resident in the household. The survey consisted of 28 questions and, based on estimates obtained during pilot work, took participants approximately 10-15 minutes to complete.

### Intervention

Devon Active Villages was designed and coordinated by Active Devon, the countywide partnership for sport and physical activity. The development of the intervention was based on the principles of community development [[28]] with rural communities encouraged to work with delivery partners, make use of local knowledge, and use local and new external resources to facilitate local involvement in the planning, promotion, and delivery of sustainable opportunities for all local people to participate in physical activity. The Devon Active Villages Evaluation (DAVE) was conducted by the University of Exeter in close liaison with Active Devon. The primary objective of the Devon Active Villages intervention was to improve participation in physical activity by offering people of all ages increased opportunities to experience the enjoyment of sport and physical activity. Therefore, examining change in physical activity prevalence between the intervention and control villages was the focus of the evaluation study. The intervention also had a number of other aims, including a target from the funder (Sport England) regarding the number of people registering to take part in Devon Active Villages activities.

The intervention was implemented and coordinated by local delivery partners, including district authority sports development teams and community-based charitable organisations. Each local delivery partner delivered the intervention in one of seven rural regions of Devon. It was necessary to have different delivery partners for each area due to the large number of villages that received the intervention in each period, and because the villages were spread across the whole county. Each delivery partner was given strategic support from Active Devon as well as a clear framework and timescales around the delivery of the intervention.

The intervention incorporated a local needs-led approach, in which each village received a ‘community engagement phase’ for twelve weeks prior to the main intervention. During this phase, delivery partners engaged with local people and community groups to carry out a needs assessment and an assessment of the activities currently on offer. This often included local people being directly surveyed to find out what activities they wanted the Devon Active Villages intervention to provide. The intervention then delivered twelve weeks of physical activity opportunities for people of all ages, with each village receiving at least three different types of activities (e.g., basketball for primary school children, multi-sports sessions for adolescents, and fitness classes for adults). Typically, activities were offered on a weekly basis over the twelve-week period. The activity sessions were subsidised using intervention funds. Delivery partners coordinated the intervention by finding suitable activity venues, purchasing necessary equipment, and hiring local experts to deliver the activities. The intervention activities were offered in cooperation with primary and secondary schools, sports clubs, and other partnerships (e.g., Sports National Governing Bodies, Premiership soccer schools). Community volunteers were recruited to help run the activities and were provided with mentoring support throughout the intervention. Intervention activities were advertised via local press (newspapers, newsletters, and radio), and with posters in local sports centres and village halls. There was no limit to the number of participants who could take part in each of the different activities. Where appropriate, delivery partners arranged additional sessions to accommodate increased demand for particular activities. Delivery partners supported the villages for twelve months following the intervention, providing them with specialist support, regular mentoring, as well as additional funding and equipment as required to help sustain the intervention activities.

### Outcomes

The primary outcome was the proportion of study participants who reported sufficient physical activity to meet the recommended physical activity guidelines, compared between the intervention and control modes as a binary outcome. A key secondary outcome was the total number of metabolic equivalent (MET) minutes per week, from which the primary outcome was derived. In addition to the above, the following outcomes were also examined: physical activity social norms, physical activity habits, perceived village supportiveness for physical activity, commitment to doing more physical activity, physical activity intentions, availability of recreational facilities in the local area, reported use of recreational facilities, and the locality of facilities used.

### Measures

#### Demographic characteristics

Participants were asked to report their gender, age, age when left full-time education, and cars in the household, based on questions from the Health Survey for England [[4]].

#### Physical activity

Physical activity was measured using the short version of the International Physical Activity Questionnaire (IPAQ-SV) [[29]]. The IPAQ-SV includes seven items on the frequency and duration of physical activities undertaken in the previous seven days (vigorous-intensity activity, moderate-intensity activity, walking, and sitting behaviour). The IPAQ-SV has been rigorously tested for test-retest reliability and criterion validity [[29],[30]].

Participants were categorised according to whether they reported sufficient physical activity to meet the current United Kingdom physical activity guidelines (at least 150 minutes of moderate-intensity activity per week in bouts of 10 minutes or more, or at least 75 minutes of vigorous-intensity activity per week) [[3]]. Physical activity level was also analysed using MET values to calculate participants’ total MET-minutes per week of moderate-intensity walking, moderate-intensity physical activity, and vigorous-intensity physical activity, using the IPAQ-SV scoring methods for calculating physical activity levels [[31]].

#### Psychosocial factors

To assess psychosocial factors, measures were created based on a multidimensional motivation for change scale [[32]], and scales developed for use in an Australian cohort study [[33]], and an English physical activity pilot programme (see List of survey measures section) [[34]]. Any negatively worded items were recoded so that higher scores were positive. Each item assessing physical activity social norms was dichotomised (“strongly disagree/disagree/neither” versus “strongly agree/agree”). The means for the ‘physical activity habits’ and ‘perceived village supportiveness for physical activity’ were taken, and the percentage of participants who scored equivalent to 1 or above (i.e., equivalent to “agree” or above) was calculated. The percentage of participants intending to do more activity within the next month or six months (as opposed to “not within the next six months” or “unlikely to ever”) was compared between the intervention and control modes. Participants’ ‘commitment to doing more physical activity’ was calculated as the mean of three constituent items, and then analysed as a continuous measure.

### List of survey measures

#### Psychosocial factors

*Physical activity social norms* (2 items – rated from -2 “strongly disagree” to +2 “strongly agree” [[32]]).

My family is interested in physical activity/sport

People around my village all seem to be exercising these days.

*Physical activity habit* (3 items - rated from -2 “strongly disagree” to +2 “strongly agree” [[32]]).

I find it easy to have a go at physical activities

I have always done some kind of physical activity

In the last 2 years, I have been involved in regular physical activity at one time or another.

*Physical activity village supportiveness* (3 items - rated from -2 “strongly disagree” to +2 “strongly agree” [[32]]).

I have recently had opportunities to get involved in physical activity.

My village is a good place to be physically active.

There are very few opportunities to be physically active in my village.

*Commitment to doing more physical activity* (3 items – rated from 0 “not at all” to 10 “very much so” [[31]]).

How important is it for you to do more physical activity than you do now?

How confident are you that you could do more physical activity if you decided to?

To what extent are you trying to do more physical activity?

*Intention to do more physical activity* (4 response items [[33]]).

I am unlikely to ever do more physical activity (1).

I intend to do more physical activity, but not in the next six months (2).

I intend to do more physical activity within the next six months (3).

I intend to do more physical activity within the next month (4).

#### Perceived local environmental characteristics

*Presence of recreational facilities within the local area* (8 items – responses 1 “yes” versus 2 “no” [[34]]).

Walking routes/footpaths

Local park/public green space

Sporting club/recreation centre/gym

River/beach/waterfront

Public swimming pool

Public tennis/squash courts

Indoor sports facility (e.g., sports hall)

Community centre/village hall

*Use of recreational facilities* (8 items – responses 0 “no, not in the last year”, 1 “yes, in last 12 months” or 2 “yes, in last month” [[32]]).

Walking routes/footpaths

Local park/public green space

Sporting club/recreation centre/gym

River/beach/waterfront

Public swimming pool

Public tennis/squash courts

Indoor sports facility (e.g., sports hall)

Community centre/village hall

*Locality of facilities used* (8 items – response box for participant to name location of facility used [[32]]).

Walking routes/footpaths

Local park/public green space

Sporting club/recreation centre/gym

River/beach/waterfront

Public swimming pool

Public tennis/squash courts

Indoor sports facility (e.g., sports hall)

Community centre/village hall

#### Perceived local environmental characteristics

Perceived proximity and use of different recreational facilities were measured using scales previously found to have acceptable test-retest reliability (Table 1) [[33],[35]]. Of the items assessing participants’ awareness of recreational facilities, only the four facilities that we would have expected to be impacted on by the intervention (‘walking routes/footpaths’, ‘local park/public green space’, ‘indoor sports facilities’, and ‘community centre/village hall’) were analysed as binary outcomes. Participants were grouped according to whether they had used at least one of the eight recreational facilities within the “last month”, in contrast to the “last 12 months” or “not at all”. Participants were also grouped according to whether they had used facilities in the “local village only” or “both inside and outside the village”, as opposed to “outside village only” or “not at all”.

**Table 1:** Sample characteristics by trial mode

**Variable**	**Trial mode**	
	**Intervention (N = 4693)**	**Control (N = 5719)**
*Male*, %	39.8	38.0
*Age in years*, mean (SD)	58.7 (15.3)	58.1 (15.3)
*Education*		
16 and under, %	36.5	38.1
17/18, %	25.8	26.3
19 and over, %	37.7	35.6
*Car ownership*		
No car	3.9	4.4
One car	37.8	39.2
Two or more cars	58.3	56.4
*Indices of multiple deprivation score* (quintiles, %)		
1 (lowest)	25.7	21.3
2	20.9	16.8
3	19.8	19.2
4	17.8	20.4
5 (highest)	15.8	22.2

#### Devon Active Villages awareness and participation

Participants were asked whether they were aware of the Devon Active Villages intervention, and if so, whether they had participated in any of its events. Participants who were aware of the intervention were also asked to select from the following response items those that most accurately reflected their opinions of the intervention: ‘I found it interesting’, ‘It’s a good campaign’, ‘It was directly relevant to me’, ‘It made me think about physical activity or exercise’, ‘It seemed irrelevant to me’, ‘It’s a waste of time’, ‘It’s a waste of money’, and ‘It had no effect on me at all’.

#### Village-level factors

Village-level factors were obtained from the 2011 Census [[36]], including percentage of villagers who were male, age classification for adult villagers, and population density. The Index of Multiple Deprivation (IMD) score was obtained at the Lower Layer Super Output Area level [[37]]. Data on the penetration of the Devon Active Villages intervention were obtained from Active Devon. Everyone who participated in the intervention was required to complete a registration form before commencing activity. From the registration details, the proportion of the population from each of the study villages attending an event was calculated, both for the whole village population and the adult population (aged 17 years or over).

### Sample size

To detect an increase from 25% to 30% of people meeting the guidelines for recommended physical activity levels, with 80% power at the 5% significance level, we recruited ten participants from each of the 128 villages at each study period. The sample size was calculated using formulae presented by Hussey and Hughes [[16]] and takes account of both within-village clustering and the number of villages receiving the intervention at each period. The intra-cluster (intra-village) correlation coefficient (ICC) for the primary outcome was assumed to be 0.02 based on published ICCs for three physical activity-related outcomes at the postcode sector level, estimated using data from the 1994 Health Survey for England [[38]].

A recent pilot for a population study of travel behaviour in the UK achieved a response rate of 20% for a short questionnaire postal survey [[39]]. On this basis, 6,400 surveys were sent out at every period (50 surveys to each village), with the expectation that at least 1,280 would be completed and returned. When this response rate was not achieved within three weeks of surveys being posted, an additional five surveys were sent out to extra households for every one survey missing. It was possible that some individuals would receive the questionnaire more than once. In such cases, if returned, demographic variables (gender, age, height, weight) were used to identify this. Recipients of the survey were made aware that their participation was voluntary; therefore informed consent was implied when participants returned a completed questionnaire. Ethical approval for this study was obtained from the University of Exeter ethics committee.

### Statistical analysis

For all outcomes, the data collected across the five periods were used in a single analysis. Analyses applied the intention-to-treat principle, with participants analysed according to the trial mode their village (cluster) was in for the period at which they provided outcome data. Unadjusted and confounder-adjusted comparisons of the outcomes between intervention and control modes were implemented using random effects (“multilevel”) linear regression, estimated using maximum likelihood [[40]] for continuous outcomes, specifying the village effect as random; and marginal logistic regression models using Generalised Estimating Equations (GEEs) with information sandwich (“robust”) estimates of standard error for binary outcomes, specifying the correlation structure as exchangeable [[41]]. The random effects model and GEEs methods allowed for the correlation between the outcomes of participants in the same village cluster, as is required for cluster randomised trials. For binary outcomes, when the intra-cluster (intra-village) correlation coefficient (ICC) was negative, instead of presenting the GEEs estimates, odds ratios from *ordinary* logistic regression were used. All analyses included period as a predictor. Adjusted models also included the following prognostic factors: region, gender, and age at the period of data collection. The ICC of the outcome was reported based on the confounder-adjusted analyses. In addition, an exploratory test of interaction was used to assess whether the effect of the intervention differed across the seven regions, a proxy for local delivery partner. All analyses were carried out using Stata software, version 12.

## Results

Of the 32,315 surveys that were sent out, 10,412 were completed and returned (response rate 32.2%, range 30.3% at wave four to 37.7% at wave one). Of these, 38.8% were male, and the mean (SD) age was 58 (15) years. Compared to the general population of the intervention villages, the study participants tended to be older (71.9% versus 59.2% aged 50 years or over), and a greater proportion were female (61.2% versus 51%). The study participants were extremely similar to the general village population in terms of their IMD scores (mean (SD) 15.8 (4.0) for both populations), and the population density of the village they resided within (mean (SD) 0.63 (0.5) for the study population versus 0.64 (0.6) for the village population). 4,693 participants provided data in the intervention trial mode and 5,719 in the control mode. The sample characteristics were similar between the intervention and control mode participants, with comparable responses being reported for gender, age, education leaving age, and car ownership (Table 1). A greater proportion of the intervention participants were in the least deprived quintile (25.7% compared to 21.3% of the control participants). More controls (22.2%) than intervention participants (15.8%) were in the most deprived quintile.

There was little evidence of an intervention effect on meeting the recommended physical activity guidelines (adjusted OR: 1.02; 95% CI: 0.88 to 1.17; p = 0.80; Table 2; Table 3), and uncertainty over the true size of the difference between intervention and control participants regarding metabolic equivalent minutes per week (adjusted mean difference: 171; 95% CI: -16 to 358; p = 0.07). At one extreme, the intervention may have had no effect on MET minutes per week, while at the other extreme it is plausible that the intervention improved physical activity levels by up to 358 metabolic equivalent minutes per week (equivalent to 90 minutes of moderate-intensity physical activity). Physical activity habits did differ between trial modes, with a greater percentage of the intervention participants having favourable activity habits than the control mode (51.5% versus 47.5%; adjusted OR: 1.18; 95% CI: 1.04 to 1.34; p = 0.009). There were no between group differences in physical activity social norms, perceived village supportiveness for physical activity, intentions or commitment to doing more physical activity, awareness of local walking routes/footpaths, local parks/public green space, indoor sports facilities or a local community centre/village hall, and use and locality of recreational facilities.

**Table 2:** Comparison of outcomes between trial modes

**Outcome**	**Trial mode**		**Crude comparison**	**Adjusted comparison (Intervention minus Control)**		
	**Intervention**	**Control**	**Statistic (I minus C)**	**Statistic (95% CI)**	**p-value**	**ICC**
Met physical activity guidelines, %	61.9	63.9	1.03	1.02 (0.88 to 1.17)	0.80	0.008
Number of metabolic equivalent minutes per week, mean (SD)	2317 (2964)	2450 (3014)	155	171 (-16 to 358)	0.07	0.010
Family is interested in physical activity (social norms), %	62.1	59.7	1.13	1.12 (0.98 to 1.26)	0.09	0.008
People around me all seem to be exercising (social norms), %	18.5	18.4	1.03	1.03 (0.87 to 1.23)	0.72	0.039
Physical activity habits, %	51.5	47.5	1.19	1.18 (1.04 to 1.34)	0.009	0.004
Perceived village supportiveness for physical activity, %	8.2	7.7	0.99	0.99 (0.78 to 1.26)	0.94	0.001
Intend to do physical activity within the next 6 months, %	61.3	57.5	0.93	0.93 (0.82 to 1.06)	0.26	0.005
Commitment to physical activity, mean (SD)	5.7 (2.6)	5.5 (2.7)	0.1	0.1 (-0.1 to 0.2)	0.33	0.006
Aware of walking routes/footpaths in the local area, %	94.0	95.0	0.95	0.89 (0.64 to 1.26)	0.52	0.029
Aware of local parks/public green space in the local area, %	80.6	78.8	1.01	1.00 (0.83 to 1.19)	0.96	0.107
Aware of indoor sports facilities in the local area, %	34.4	32.9	1.00	0.97 (0.86 to 1.10)	0.62	0.260
Aware of community centre/village hall in the local area, %	83.9	80.9	1.02	0.97 (0.80 to 1.19)	0.80	0.095
Used recreational facilities within the last month, %	84.9	85.2	0.97	0.94 (0.78 to 1.13)	0.49	0.024
Used at least one recreational facility in the village, %	71.3	72.5	0.96	0.94 (0.82 to 1.09)	0.42	0.084

The trial mode statistics are the mean scores (or overall percentage) within the mode across all five periods (stages). Note that because all comparisons are adjusted for period the direction of effect does not necessarily correspond with the within mode summary statistics. A detailed breakdown of results within each period is shown in Table 3 for ‘*Meets physical activity guidelines’* and ‘*number of MET minutes per week’*.

The comparative statistic is the Mean Difference for quantitative outcomes, and the Odds Ratio for dichotomous outcomes. Sample size ranged from 3892 to 4693 in the intervention mode and 4657 to 5719 in the control mode. Crude analyses adjusted for period. Adjusted analyses adjusted for period, gender, age, and area.

**Table 3:** Crude comparison of physical activity variables by period

		**Trial mode**	
**Period**		**Intervention**	**Control**
1	N	-	*2,409*
	Meets physical activity guidelines, %	-	66.9
	Number of MET minutes/week, mean (SD)	-	2561 (2977)
2	N	*312*	*1,625*
	Meets physical activity guidelines, %	67.3	61.5
	Number of MET minutes/week, mean (SD)	2848 (3191)	2449 (3109)
3	N	*921*	*1,082*
	Meets physical activity guidelines, %	60.0	58.8
	Number of MET minutes/week, mean (SD)	2304 (3033)	2137 (2956)
4	N	*1,380*	*522*
	Meets physical activity guidelines, %	64.6	68.2
	Number of MET minutes/week, mean (SD)	2512 (3084)	2585 (2961)
5	N	*1,971*	-
	Meets physical activity guidelines, %	60.1	-
	Number of MET minutes/week, mean (SD)	2101 (2785)	-
*Total*	N	*4,584*	*5,638*
	Meets physical activity guidelines, %	61.9	63.9
	Number of MET minutes/week, mean (SD)	2317 (2964)	2450 (3014)

*N* – sample size.

There was little evidence that the effect of the intervention on meeting the recommended physical activity guidelines was modified by study area (interaction test p = 0.62). Post-hoc analyses also showed there was little evidence that the intervention had a delayed effect (p = 0.79) or an immediate effect that subsided (p = 0.98).

Of the study participants in the intervention mode 16% reported awareness of Devon Active Villages, and 4% reported participation in intervention events (Table 4). Of those reporting awareness of the intervention, 50.6% agreed it was a good campaign, 29.8% found the intervention interesting, and 25.1% reported that the intervention made them think about physical activity or exercise. In total, 80% of the opinions on the Devon Active Villages intervention were positive.

**Table 4:** **Participation and opinions on the DAV intervention**^**†**^

**Participation/opinion**	**%**
Participated in the DAV intervention	25.0
Opinions on the DAV intervention:	
*I found it interesting*	29.8
*It’s a good campaign*	50.6
*It was directly relevant to me*	16.2
*It made me think about physical activity or exercise*	25.1
*It seemed irrelevant to me*	7.4
*It’s a waste of time*	1.2
*It’s a waste of money*	2.6
*It had no effect on me at all*	13.0

†Sample size is the 745 (16.0%) participants from the intervention mode who were aware of the DAV intervention.

### Intervention registrations

In the intervention villages, 5.2% of the population registered to participate in Devon Active Villages events (Table 5), although when children (aged 16 years and under) were excluded, this figure was reduced to 2.7%. The greatest participation in Devon Active Villages activities occurred in the villages that received the intervention in the third time period for the adult population (4.3%). Several villages failed to participate in the intervention, while others achieved up to 48% population penetration. At the time data collection was concluded for this study, the wider Devon Active Villages intervention was ahead of target for the number of people registered for Devon Active Villages activities. No formal analysis was possible for this measure as the data were not collected in a systematic way across the programme.

**Table 5:** Proportion of the population of study villages that registered as participants in the ‘Devon Active Villages’ intervention

**Batch**^**†**^**(Period in which intervention was first received)**	**% total population Median (range)**	**% 17+ years population Median (range)**
1 (Period 2)	8.3 (0 to 24.8)	3.9 (0 to 20)
2 (Period 3)	6.9 (0 to 48)	4.3 (0 to 17.7)
3 (Period 4)	4.8 (0 to 19.2)	1.4 (0 to 13.2)
4 (Period 5)	3.9 (0 to 23.6)	1.0 (0 to 8.3)
*Overall*	5.2 (0 to 48)	2.7 (0 to 20)

^†^Each batch contains the villages that first received the intervention in the same specified period.

The village is the unit of analysis.

## Discussion

The aim of this study was to evaluate the effectiveness of Devon Active Villages, a community-level physical activity intervention delivered to rural villages. The Devon Active Villages intervention had no effect on the proportion of people active at recommended levels, and there was uncertainty regarding the true size of the increase in the number of MET-minutes per week reported, as reflected in the 95% confidence interval for the mean difference. It is possible that the intervention was effective at the individual level, but the low levels of population penetration prevented any observable effect at the village level.

Ensuring sufficient penetration and reach across a community to attain a population-level impact is one of the most difficult aspects of community-level interventions [[10]]. Although few studies have reported population participation rates, one review found that the highest exposures were obtained for public information and screening activities rather than more intensive interventions, and that population penetration rates ranged from 4-60% [[10]]. In the Devon Active Villages intervention, there was only a limited budget for promotion activities, which may have contributed to the low levels of participant awareness in the research study. However, in rural areas with an ageing population, it is arguably more difficult to find effective ways of communicating new physical activity opportunities to sedentary individuals, because most methods rely on participants seeing an advertisement in the local area. Media activities (e.g., television, radio) can achieve greater levels of reach, but can also be expensive for localised community-based interventions, such as the Devon Active Villages intervention. Despite the intervention incorporating a local needs-led approach, the budget only allowed for 1-2 activities per age group per village. Thus, it is possible that the provided activities did not appeal to all residents who were aware of the intervention.

Baker et al. [[11]] conducted a systematic review of community-level physical activity interventions and found that only three out of the 25 included studies reported positive changes in physical activity behaviour [[42]–[44]]. Jiang et al. [[42]] conducted an intervention in urban communities within Beijing, finding a reported increase in regular physical activity in the intervention group (adjusted relative risk 1.20, 95% CI 1.09 to 1.31). However, the intervention achieved substantial penetration within the community (73% participation), through ‘door-to-door’ hand-outs and individualised counselling by health practitioners. In the Finnmark Intervention study [[43]], a sport and activity-based intervention in a small artic community in Norway, males reported a significant increase (p = 0.047) in physical activity behaviour six years after the initial baseline measurement. No change was found in the female population, however. Similar to the Beijing study, the Finnmark Intervention reached large segments of the population, through community engagement, mass media, and individual counselling. The only other study in the review to find an increase in physical activity was the Rockhampton 10,000 Steps Project [[44]], where the proportion of females who met the recommended guidelines increased significantly from baseline to post-intervention. The study found no evidence of physical activity behaviour change in males. Again this intervention involved a large number of components, including social marketing, pedometers, individual counselling, partnering with local organisations, and environmental changes.

In contrast, the studies that reached a smaller proportion of the population, either through low cost or low activity, found no intervention effect on physical activity [[11]]. For example, the low cost of one intervention in rural municipalities in Denmark limited the amount of intervention activities that took place, resulting in the intervention being purely mass-media [[45]]. Simon et al. [[46]] was one example of a low reach intervention, aimed at school communities in France. Although the intervention initially aimed to reach the whole community, in actuality, the vast majority of the intervention activities were targeted at one specific section of it. This was similar to Devon Active Villages, where many of the intervention activities were targeted at a specific group within the community (i.e., basketball for primary school children, or armchair aerobics for older adults). From the population penetration rates achieved by Devon Active Villages, it is clear that the intervention would be classed as ‘low reach’. Therefore, the results of the present investigation are in line with previous research, where interventions with low reach failed to have an effect on physical activity behaviour [[11]].

Despite the above, the intervention was associated with stronger activity habits, suggesting that those in the intervention mode perceived themselves to be physically active, but did not report a greater level of physical activity than controls. Physical activity habits was the only outcome for which there was evidence of an effect. We are not aware of any other community interventions that have reported physical activity habit as an outcome.

The majority of reported intervention opinions were positive, suggesting that the intervention was well received by the small proportion of participants who were aware of its existence.

### Strengths and limitations

Strengths of the study include the large sample size (>10,000) and the large number of participating villages. Incorporating multiple data collection periods into the research meant that it was possible to analyse both whether the intervention had an immediate effect on physical activity that later subsided, or whether the intervention effect was delayed. Each village acted as its own control, meaning communities were not subjected to “best-fit” matching with control communities. Another strength is that the period in which villages first received the intervention was randomly allocated, eliminating any selection bias. Indeed, in a recent review of community-level physical activity interventions [[11]], only one study out of 25 used randomisation to allocate communities [[46]].

This study fills a gap in the literature by being the first to use a stepped wedge cluster randomised trial design to evaluate a physical activity intervention. Examples of previous stepped wedge investigations include examination of the efficacy of Hepatitis B vaccinations [[47]], the effect of housing improvements on respiratory health symptoms [[48]], and different tuberculosis treatments on number of disease episodes [[49]]. The stepped wedge trial design was the most appropriate study design for this intervention for three reasons: first, there was a necessity to deliver the intervention in waves due to limited resources; second, once the intervention was implemented it was never fully taken away; and third, the intervention was delivered to all eligible communities of a certain size within the county [[16]]. Despite the stepped wedge trial design requiring greater data collection and longer trial duration [[16]], it was successfully able to evaluate a pragmatic community-level physical activity intervention.

Despite being better than anticipated, and comparing well with other survey studies from the United Kingdom (15.9% [[50]], 17% [[39]]), the response rate was low (32.2%). Non-response bias often occurs in survey studies, where non-responders may differ in some way from those who do respond [[51]]. The participants in the present research were similar to the wider population in terms of IMD score and the population density of the village they resided in. Compared to the wider population, however, the survey respondents tended to be older, with a greater proportion being female. Previous research suggests females and older adults are often over-represented in health surveys [[4]]. Survey respondents also tend to report being healthier and doing more physical activity than the general population [[52]]. Two-thirds of the present research population reported meeting the recommended guidelines, suggesting that those of higher activity levels were over-represented. However, previous research suggests that the IPAQ-SV has a tendency to over-report time spent doing physical activity [[53]–[55]], with one review finding that the IPAQ-SV over-reported physical activity on average by 106% (Range 36-173%) [[55]]. Nevertheless, if the more physically active are over represented in the study it could be that the intervention effect is smaller for these people than those who did not respond and who might not normally engage with physical activity.

Participants may have over-reported exposure to the Devon Active Villages intervention events because they believed this response to be favourable to the researchers [[49]]. However, the high level of consistency between the reported participation and participation according to village registrations suggests that such reporting bias was not present in this study. In addition, while the generally positive intervention opinions may have been an accurate representation of how well the intervention was received, participants may have reported overly positive opinions in an attempt to stop any intervention funding from being withdrawn [[51]].

The main limitation of this research is the use of self-reported data. Self-reported outcome measures of physical activity tend to include bias due to social desirability and may lead to some misclassification, with some participants finding it difficult to recall activities from the past seven days. Nevertheless, there is no reason to believe that any misclassification was systematically different with regard to intervention or control group. Furthermore, established and validated measures were used where possible (e.g., the IPAQ-SV to measure physical activity). Another potential limitation is the nature of the study sample. Although the intervention was available to all age groups, the study focused on adults, because child and youth physical activity comprises a separate body of literature with different guidelines and understanding about what constitutes physical activity behaviour in this age group.

Repeated cross-sectional samples of participants were used in this research in order to measure the community-level impact of the intervention on physical activity levels, rather than follow individuals over time to detect individual changes in behaviour. Although it is possible that the repeated cross-sectional samples included people new to the village who were not exposed to the intervention, it is perhaps more likely that there was contamination due to people in control villages participating in neighbouring village intervention activities. Both of these factors would have attenuated intervention effects [[10]]. Finally, it may be that the reach, intensity and duration of the intervention were insufficient to achieve a population-level impact.

### Implications

The results of this research indicate that unless community-level physical activity interventions can reach a substantial proportion of the target population they are unlikely to be able to change the population prevalence of physical activity. This research also demonstrated that it is possible to rigorously evaluate pragmatic community-level physical activity interventions using novel research techniques. This research is also the first to use a stepped wedge cluster randomised trial design to evaluate a community-level physical activity intervention. The stepped wedge design was suitable for evaluating the Devon Active Villages intervention, because it was by necessity delivered in waves, administered to all eligible communities in the population, and, once a community received the intervention, it was never fully taken away. This study also adds to the limited research available on physical activity in rural communities from England.

### Future research

It is advocated that future evaluation studies consider the use of the stepped wedge cluster randomised trial design for evaluating health interventions, especially for community-level physical activity interventions. Additionally, more rigorous evaluations of community-level physical activity interventions are needed to help understand what works in altering population prevalence. In order to improve validity and reliability, these intervention evaluations should include objective measurements (e.g., accelerometry data). Finally, more research is warranted on how to achieve greater community penetration/engagement in community-level physical activity interventions.

## Conclusions

An experimental approach to the design and evaluation of the Devon Active Villages intervention showed no evidence that the intervention increased the prevalence of physical activity within the villages, and only weak evidence of an increase in physical activity level. The intervention did lead to an increase in physical activity habits. The evaluation highlighted that very few residents were even aware of and participated in the intervention. Evaluating population-level interventions is challenging but not impossible. Better understanding of the effectiveness of such interventions will only be achieved if more community-level interventions, which continue to be funded, are evaluated with more robust research designs. Future interventions need to both deliver effective interventions and achieve a high level of reach to achieve changes in population prevalence.

## Abbreviations

CI: Confidence interval

DAVE: Devon Active Villages Evaluation

GEE: Generalised Estimating Equation

ICC: Intra-cluster correlation coefficient

IMD: Indices of multiple deprivation

IPAQ-SV: International Physical Activity Questionnaire – Short Version

MET: Metabolic equivalent

N: Number

OR: Odds ratio

SD: Standard deviation

## Competing interests

The authors declare that they have no competing interests.

## Authors’ contributors

ES designed the study and data collection tools, obtained ethics approval, acquired funding, carried out the data collection, cleaned and analysed the data, drafted and revised the paper. ES is guarantor and responsible for the overall content of the manuscript. TR identified the research question, designed the study, acquired funding, and revised the draft paper. OCU fine-tuned the research methodology, conducted the randomisation procedures, analysed the data, and revised the draft paper. BM analysed the data, and revised the draft paper. MH identified the research question, designed the study, acquired funding, analysed the data, and drafted and revised the paper. All authors read and approved the final manuscript

## Authors’ information

Note: The submitted research was conducted at the University of Exeter while Emma Solomon (the guarantor) was a PhD researcher. Emma Solomon is now a Research Associate at the University of the West of England, thus up-to-date contact details have been provided.
